# Circulating microparticle proteins predict pregnancies complicated by placenta accreta spectrum

**DOI:** 10.1038/s41598-022-24869-0

**Published:** 2023-01-05

**Authors:** Hope Y. Yu, Serena B. Gumusoglu, David E. Cantonwine, Daniela A. Carusi, Prem Gurnani, Brandon Schickling, Robert C. Doss, Mark K. Santillan, Kevin P. Rosenblatt, Thomas F. McElrath

**Affiliations:** 1 Division of Maternal-Fetal Medicine, Department of Obstetrics and Gynecology, Brigham and Women’s Hospital, Harvard Medical School, Boston, MA USA; 2 University of Iowa Carver College of Medicine, Iowa City, IO USA; 3 NX Prenatal Inc., Louisville, KY USA; 4 Division of Oncology, Department of Internal Medicine, University of Texas Health Science Center at Houston, McGovern Medical School, Houston, TX USA

**Keywords:** Biomarkers, Predictive markers

## Abstract

Placenta accreta spectrum (PAS) is characterized by abnormal attachment of the placenta to the uterus, and attempts at placental delivery can lead to catastrophic maternal hemorrhage and death. Multidisciplinary delivery planning can significantly improve outcomes; however, current diagnostics are lacking as approximately half of pregnancies with PAS are undiagnosed prior to delivery. This is a nested case–control study of 35 cases and 70 controls with the primary objective of identifying circulating microparticle (CMP) protein panels that identify pregnancies complicated by PAS. Size exclusion chromatography and liquid chromatography with tandem mass spectrometry were used for CMP protein isolation and identification, respectively. A two-step iterative workflow was used to establish putative panels. Using plasma sampled at a median of 26 weeks’ gestation, five CMP proteins distinguished PAS from controls with a mean area under the curve (AUC) of 0.83. For a separate sample taken at a median of 35 weeks’ gestation, the mean AUC was 0.78. In the second trimester, canonical pathway analyses demonstrate over-representation of processes related to iron homeostasis and erythropoietin signaling. In the third trimester, these analyses revealed abnormal immune function. CMP proteins classify PAS well prior to delivery and have potential to significantly reduce maternal morbidity and mortality.

## Introduction

Placenta accreta spectrum, or PAS, is characterized by aberrant placentation ranging from abnormal adherence to invasion through the uterine wall and into neighboring pelvic structures^1^. The central pathogenic feature of PAS is that the placenta does not spontaneously separate from the uterus after delivery of the fetus. Therefore, PAS may lead to catastrophic obstetric hemorrhage, emergent hysterectomy, multiorgan failure, and death^2^. As such, PAS is a significant contributor to maternal morbidity and mortality around the world, and its incidence is increasing^3,4^. Reliable antenatal identification of PAS would allow for transfer to PAS referral institutions where appropriate multidisciplinary delivery care can be provided. Appropriate care at such institutions reduces maternal morbidity from PAS by up to 80%^5,6^. However, up to half of PAS is undiagnosed prior to delivery^7–9^. Therefore, improving early- and mid-gestation identification of PAS is critical to the global effort to minimize maternal morbidit ^5–7^.

Currently, a woman’s risk for PAS is assessed using a combination of clinical/historical risk factors followed by radiologic evaluation with either ultrasound or ultrasound in combination with magnetic resonance imaging (MRI). The most common risk factors for PAS are placenta previa and prior cesarean delivery^10^. This diagnostic paradigm is, however, insensitive, as one-third to one-half of PAS cases remain undiagnosed prior to delivery, and this strategy performs poorly among patients without identified clinical risk factors^7–9,11^. Given these limitations, a readily accessible PAS biomarker would be of unparalleled clinical utility to inform a woman’s risk of PAS, particularly in low resource and rural settings without specialized ultrasound services. Identifying a PAS biomarker would also reveal mechanistic and potentially therapeutic insights into this disease.

Circulating microparticles (CMPs) are nano- to micro-sized lipid bilayer extracellular vesicles found in all intracellular media that mediate cellular signaling and intercellular communication^12^. CMPs have been utilized extensively in oncology biomarker research and have shown promise in obstetrics research, particularly in preterm birth and preeclampsia^13–17^. CMP contents also offer a targeted glimpse into the state of cellular crosstalk, thereby exposing the autocrine and paracrine factors involved in trophoblastic invasion at the maternal–fetal interface in PAS. With the dual goals of identifying a clinically useful PAS biomarker, as well as identifying CMP mechanisms of PAS pathogenesis, we conducted a nested case–control study to identify CMP-associated protein panels that identify pregnancies complicated by PAS.

## Results

### Clinical characteristics of PAS cases

Maternal plasma samples were collected in the second and third trimesters at a median 26 (± 2) and 35 (± 2) weeks’ gestation, respectively. 35 PAS cases and 70 controls were analyzed including 27 cases of grade 1 PAS, 7 cases of grade 2 PAS, and 1 case of grade 3 PAS as defined by the International Federation of Gynaecology and Obstetrics (FIGO) (Ref.^18^; Table 1). Compared to controls, cases were more likely to be of older maternal age and have placenta previa at delivery. Twenty (57.1%) of the PAS cases did not have a history of cesarean section nor placenta previa at delivery. Twenty-three (65.7%) of the PAS cases were determined by clinical criteria rather than histologic criteria. Notably, only 11% of grade 1 PAS cases were detected by ultrasound prior to delivery, compared to 57% and 100% of grade 2 and 3 PAS cases, respectively (Table 2).

**Table 1:** FIGO clinical and histologic criteria for PAS. Adapted from Jauniaux et al.^18^.

	Clinical criteria	Histologic criteria
Grade 1	Attempts at manual removal of adherent placenta results in heavy bleeding requiring mechanical or surgical measures	Hysterectomy specimen with extended areas of absent decidua between villous tissue and myometrium
Grade 2	Macroscopic findings: hypervascularity, placental bulge, “dimple sign”	Hysterectomy specimen shows placental villi within muscular fibers
Grade 3	Macroscopic findings of Grade 2 **AND** placental villi seen to be invading through uterine surface ± invasion into other pelvic structures	Hysterectomy specimen showing villous tissue breaching the uterine serosa

**Table 2:** Characteristics and grading determination of PAS cases. ^a^Data displayed as median (± IQR) or n (%).

	Cases^a^ n = 35	Controls^a^ n = 70	p-value
Maternal age (years)	36.9 (± 2.9)	32.9 (± 2.7)	< 0.01
**# prior cesarean sections**			
0	23 (65.7)	55 (78.6)	0.33
1	7 (20.0)	10 (14.3)	
≥ 2	5(14.3)	5 (7.1)	
Placenta previa at delivery	8 (22.9)	6 (8.6)	< 0.01
**PAS diagnosed by ultrasound**			
Grade 1 (n = 27)	3 (11.1)	N/A	
Grade 2 (n = 7)	4 (57.1)		
Grade 3 (n = 1)	1 (100.0)		
**PAS grade and determination**			
**Grade 1**	27 (77.1)	N/A	
Clinical	21		
Histologic	0		
Both concordant	6		
**Grade 2**	7 (20.0)		
Clinical	2		
Histologic	1		
Both concordant	4		
**Grade 3**	1 (2.9)		
Clinical	0		
Histologic	0		
Both concordant	1		

### Putative CMP protein panels and performance characteristics

**Figure 1:**
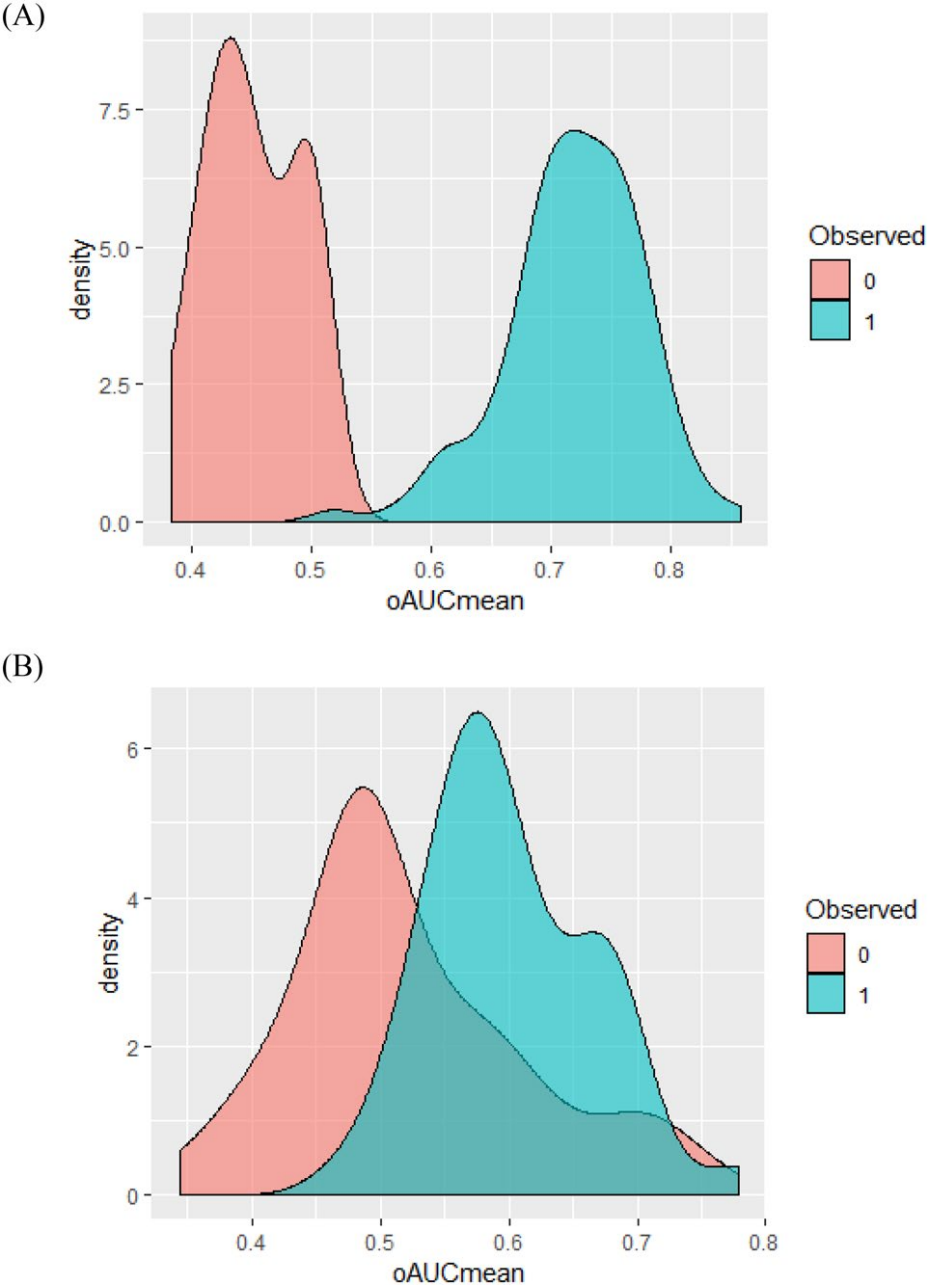
Density plots of protein versus permuted. Blue shaded area represents actual protein AUC. Red shaded area represents AUC from randomly permuting the sample labels (PAS vs. control). (**A**) Second trimester, (**B**) Third trimester.

To establish a panel of CMP proteins that would serve as a classifier for the risk of PAS in the second and third trimesters, we implemented a two-step iterative workflow using regularized (L1) regression followed by a cross-validation procedure using logistic regression. The two-step workflow was also repeated with randomly permuted sample labels to simulate random chance. In second trimester samples, the mean of all area under the curves (AUCs) for the observed versus permuted panels was significantly different (0.72 vs. 0.45; p < 2.20e^−16^; Fig. 1A). The top performing panel of markers distinguished PAS from controls with a mean AUC of 0.83. CMP proteins of this panel included: isthmin-2 (ISM2), sulfhydryl oxidase 1 (QSOX1), histone H4 (H4), hemoglobin subunit gamma-2 (HBG2) and cartilage acidic protein 1 (CRAC1). Within third trimester samples, the mean of all AUCs for the observed versus permuted panels were also significantly different (0.60 vs 0.52; p = 2.79e^−5^; Fig. 1B). The top performing panel distinguished PAS from controls with a mean AUC of 0.78. CMP proteins in this panel included ISM2, ubiquitin carboxyl-terminal hydrolase 1 (UBP1), immunoglobulin lambda variables 10–54 (LVX54) and Ig-like domain-containing protein. Additionally, the mean of all AUC in the second trimester was significantly greater than that in the third trimester (p = 1.59e^−12^).

### T2 canonical pathway, upstream regulators, and molecular and cellular function analyses

To determine the biological function of those proteins identified as differentially expressed by PAS status in our differential expression analysis, we also applied Ingenuity Pathway Analysis (IPA, version 01-20-04). This revealed significant over-representation of canonical pathways, upstream regulators, and molecular and cellular functions. In the second trimester, proteomic changes in PAS yielded significant over-representation of several canonical pathways including iron homeostasis signaling and erythropoietin signaling (Table S1). Master upstream regulators included seven targets predicted to be significantly activated and six predicted to be significantly inhibited (Table S2). IPA molecular and cellular functions analyses revealed 43 select annotated functions which were significantly over-represented based on differentially-expressed molecular hits from second trimester placenta accreta analyses (Table S3). Cellular and molecular functions around iron handling and erythrocyte function agreed with canonical iron homeostasis and erythropoietin signaling pathways.

### T3 canonical pathway, upstream regulators, and molecular and cellular function analyses

As with second trimester data, IPA Core Analysis revealed significant over-representation of canonical pathways, upstream regulators, and molecular and cellular functions in the third trimester in PAS. Canonical pathway analysis of third trimester proteomic changes in PAS revealed significant over-representation of pathways including immune and extracellular signaling pathways, specifically involving IL-15 (Table S4). Master upstream regulators included three predicted to be significantly activated and two significantly inhibited (Table S5). Molecular and cellular functional analyses revealed 24 select annotated functions which were significantly over-represented based on differentially-expressed molecular hits from third trimester analyses in PAS (Table S6). There was some agreement, particularly around immune signaling and cytoskeletal and cell growth functions, between these cellular and molecular functions and the canonical pathways revealed by IPA.

## Discussion

We demonstrate that a panel of five CMP proteins classifies PAS in the second trimester, and a panel of four proteins classifies PAS in the third trimester, both with a degree of accuracy that implies clinical utility. This finding is of high potential value for the clinical diagnosis and management of PAS. It also offers critical insights into underlying pathogenesis. While the pathophysiology of PAS remains unclear, it is likely that aberrations in trophoblast invasion, apoptosis, and angiogenesis are involved^19,20^. ISM2 was the only protein common to both the second and third trimester panels. Isthmins, including ISM2, represent a family of secreted proteins with diverse functions including cell adhesion and angiogenesis. ISM2 is expressed at high levels in the placenta and is overexpressed in cases of choriocarcinoma, consistent with the invasive nature of PAS^21^. QSOX1, another PAS marker revealed here, has also been implicated in tumor cell invasion, through activation of MMP-2 and MMP-9^22^. This is consistent with prior PAS studies that attribute placental invasion to activation of MMPs^23^. Additional proteins identified on the PAS panels described here may also reveal mechanisms of PAS pathogenesis. For instance, H4 has been implicated in apoptosis and is an early marker of cell death^24^. HBG2 comprises fetal hemoglobin, the breakdown products of which are associated with increased oxidative stress^25^. CRAC1 is an extracellular matrix protein associated with matrix disorders and involved in cell proliferation, regeneration and collagen degradation^26^.

Our results also suggest a greater predictive value of second trimester markers relative to third trimester markers. Proteins within the second trimester panel are more specific to cellular invasion and angiogenesis functions than those in the third trimester panel. This difference may be due to the unique state of placental growth and development at the end of the second trimester. For example, the final phase of villous blood vessel growth occurs at 24–26 weeks’ gestation, leading to exponential growth of the terminal villi and an increase in placental blood flow. This may contribute to increased CMP trafficking and therefore more predictive disease signal^27^. Clinically, the second trimester timepoint conveniently corresponds with a routine clinical blood draw for gestational diabetes screening, and therefore, our findings could be easily adapted into a clinical screening regimen^28^. Furthermore, identification of PAS at an earlier gestational age allows ample time for patient counseling and optimization of multidisciplinary care.

**Figure 2:**
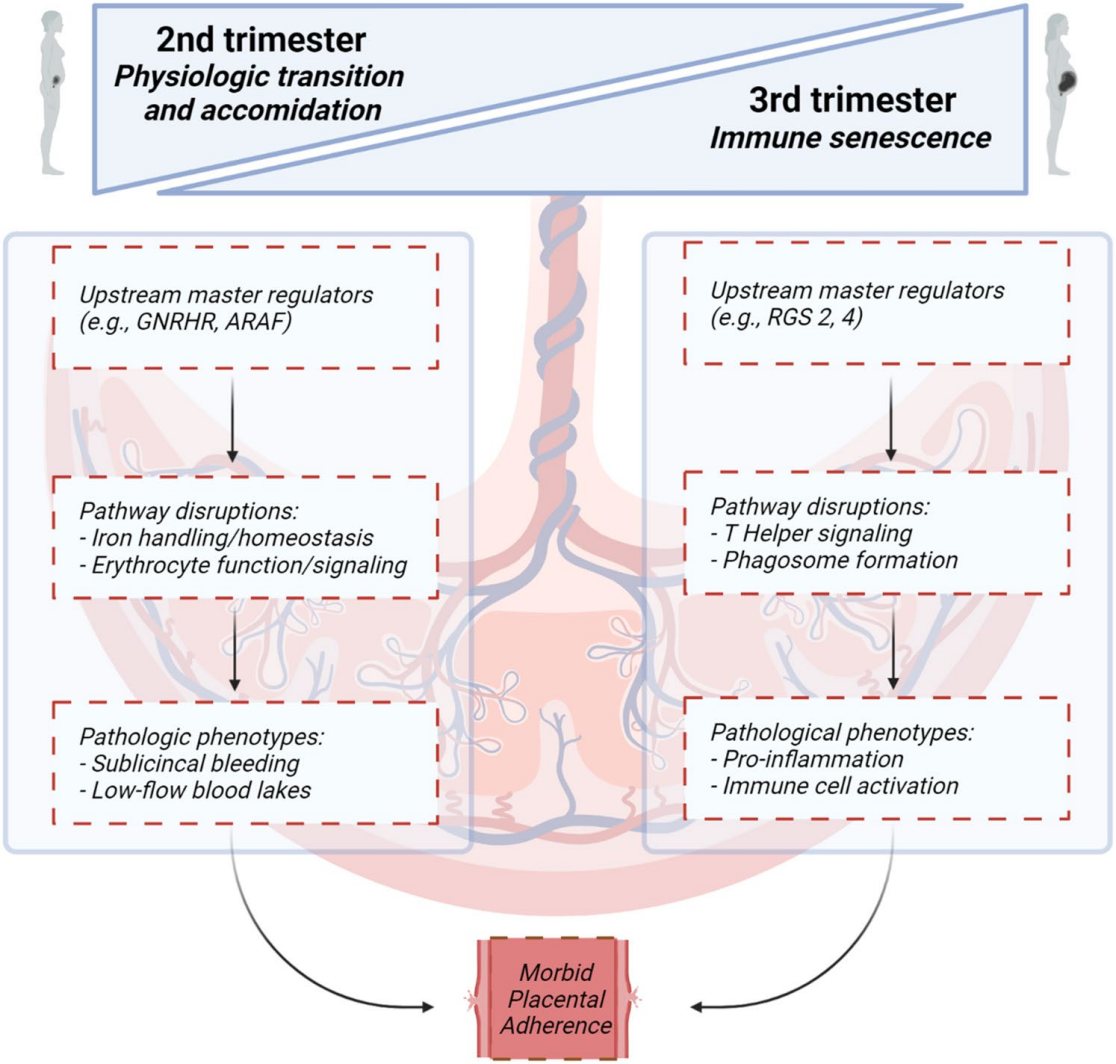
Canonical pathways, upstream regulators and molecular and cellular function analyses proposed in the second and third trimester leading to morbid placental adherence. Figure created with BioRender.com.

Additionally, analyses of proteomic signatures from our matched second and third trimester samples reveal processes underlying the pathophysiology of PAS (Fig. 2). In the second trimester, canonical pathway analyses demonstrate over-representation of processes related to blood cell function and oxygenation, including iron homeostasis and erythropoietin signaling. Dysregulation in these processes may indicate subclinical bleeding evidenced by presence of placental lakes on ultrasound, inflammation, and/or anemia in second trimester PAS. GNRHR and ARAF are two upstream master regulators that we identified and have been previously linked to processes impacting PAS development, specifically regulation of trophoblast invasion and endometrium decidualization^29–34^. In the third trimester, similar analyses characterized abnormal innate and adaptive immune cell and IL-15 signaling. Immunoglobulins and complement factors are involved in multiple implantation and placentation processes which may be abnormal in PAS^35,36^. RGS-family proteins and ADCY are master regulators of third trimester PAS proteomics that are known mediators of immune function. RGS-family proteins have well-defined roles in cellular immunity, T cell function, and physiologic inflammation and immunity processes, with RGS2 being a known placental mediator in preeclampsia^37–39^. ADCY has similarly been implicated in inflammatory processes and mechanisms which might underlie PAS pathogenesis, including endothelial inflammation mechanisms^40^ and trophoblast syncytialization processes^41^.

Our study stands out from prior PAS biomarker studies for several reasons. First, inclusion into our PAS cohort did not require antenatal diagnosis or suspicion by ultrasound; an inclusion criterion common to other studies^42–45^. Importantly, 77% of our PAS cases were not detected antenatally by ultrasound. A large proportion of our PAS cases were Grade 1 which had a startlingly low detection rate of 11% (in contrast to a 57% and 100% detection rate for Grade 2 and Grade 3 cases, respectively). Second, our PAS cohort represents the entire spectrum of disease whereas prior studies limited their cohorts to the most severe cases or required the presence of risk factors such as placenta previa and prior cesarean delivery^45–49^. We intentionally cast a wide net for potential cases while rigorously assessing each to ensure less invasive cases were included but remained on the disease spectrum of PAS per FIGO criteria. While an argument could be made that Grade 1 cases carry less morbidity than Grade 2 or 3 cases, this is likely to only be true when there is high antepartum clinical suspicion. Grade 1 cases can result in significant maternal morbidity when unidentified and managed without proper hospital resources and delivery preparation. Therefore, our detection of a high-performing biomarker panel in this cohort has promising potential to improve antenatal detection of even the most elusive PAS cases and ensure appropriate preparations can take place prior to delivery.

This study needs to be interpreted within the context of its design. While our sample size is equivalent to existing PAS biomarker studies, we set the upper limit of panel size to five to avoid over-subscribing our data. Additionally, we acknowledge that clinical factors or ultrasound findings may further improve the AUC in larger datasets, and future studies should evaluate how addition of a biomarker can improve the current diagnostic paradigm. Ultimately, these findings will need to be replicated in an independent cohort. Our study offered the advantage of limited subjectivity, as independent reviewers graded PAS cases, with discrepancies adjudicated by a third review. Importantly, this is also the first study to use CMP proteins to classify PAS. This specific, targeted assessment allowed us to evaluate the biomarkers most representative of cellular activity and communication. In addition to advancing the mechanistic understanding of PAS and search for treatments, it is our hope that these findings will be used clinically to identify pregnancies affected by PAS and ultimately to improve maternal outcomes.

## Methods

### Sample collection

We performed a nested retrospective 1:2 case–control study selected from the on-going LIFECODES pregnancy biobank at Brigham and Women’s Hospital, Boston, MA. Samples were collected over the time period of 2007–2020. Methods pertaining to patient recruitment, sample collection and clinical data curation for LIFECODES are detailed in our prior publications and summarized here^15,16,50^. Patients were approached at their first prenatal visit. Eligibility criteria included patients who were > 18 years of age, receiving prenatal care and planning on delivery at Brigham and Women’s Hospital. After written informed consent was obtained, EDTA plasma samples were obtained, aliquoted, and stored at − 80° centigrade. Pregnancy dating was confirmed by ultrasound at ≤ 12 weeks gestation. If consistent with the last menstrual period (LMP) dating, the LMP was used to determine the due date. If not consistent, then the due date was set by the earliest available ultrasound ≤ 12 weeks gestation. The medical record for each subject in the LIFECODES biobank is independently reviewed by two Maternal–Fetal Medicine faculty physicians. Complications and outcomes for each subject are coded using a structured coding tool. The codes from each reviewer are then compared with disagreement in either pregnancy outcome or complication is adjudicated by a review committee. The biobanking and research protocol were approved by the institutional review board at Brigham and Women’s Hospital and Mass General Brigham. All methods were performed in accordance with relevant guidelines and regulations.

We defined cases as subjects with clinical or histologic grade 1–3 PAS consistent with the 2019 FIGO PAS classification (Ref.^18^, Table 1*)*, delivery ≥ 23 weeks gestation and inclusion in the LIFECODES biobank. Prospective cases were first identified in the electronic medical record using the following word searches for records between 2007 and 2020: “adhere-” in operative reports and discharge summaries as well as “-creta,” “hyst-” or “previa” in pathology reports. The medical record for each prospective case was independently reviewed by two obstetricians within our institution’s multidisciplinary PAS team. The higher grade between the clinical and histologic grades was designated as the assigned grade. Disagreement in either inclusion status or assigned PAS grade were adjudicated by a review committee. Subjects identified to have a PAS diagnosis were cross-referenced with the LIFECODES biobank for inclusion. Controls were defined as subjects without a diagnosis of PAS and randomly matched 2:1. Cases and controls were matched by gestational age of sampling (± 1 week) and number of fetuses. We defined exclusion criteria as current cancer diagnosis, use of immunomodulating medication or documented fetal chromosomal abnormality. Univariate analyses were conducted with chi-square tests and continuous variables were compared with Wilcoxon tests using SAS 9.4. All tests were two-tailed; *P* < 0.05 was used to define statistical significance.

### CMP enrichment

We utilized Size Exclusion Chromatography (SEC) for CMP isolation. Our methods have been detailed in our prior publications^15,16,50,51^. SEC has been evaluated and reviewed favorably as an efficient means for microparticle isolation^52^. Anonymized EDTA plasma samples identified only by a study number that was agnostic to case or control status were randomly assorted and shipped on dry ice to NX Prenatal, Inc. (Houston, TX) where CMP protein enrichment was carried out by SEC and isocratically eluted using the NeXosome^®^ Elution Reagent. This involved NeXosome^®^ Isolation Columns manufactured by AmericanBio, Inc. (Canton, MA). These columns were packed by AmericanBio with Sepharose 4B-CL (4% agarose, particle size 45–165 μm) from Cytiva (Marlborough, MA) to a total packed volume of 10 mL and delivered to NX Prenatal. Once received by NX Prenatal, the columns were stored at 2–8 °C until use. Prior to using the columns for CMP isolation, they were allowed to equilibrate to room temperature (overnight) and subsequently washed with NeXosome^®^ Elution Regent. EDTA plasma samples were thawed and 0.5 mL of plasma was applied and allowed to incorporate into the NeXosome^®^ Isolation Column. The plasma samples were not filtered, diluted, or pretreated prior to application to the columns. Following the incorporation of the sample into the column, the NeXosome^®^ Elution Reagent was added and 0.5 mL column fractions were collected. As previously published, the eluted fractions yielded two peaks. The CMPs were captured in the column void volume and resolved from the high abundant soluble protein peak^51^. Samples were processed according to the randomization scheme provided by Brigham and Women’s. Each CMP-containing fraction (0.5 mL aliquots of each fraction) was pooled within each individual sample and a total protein measurement was performed, using the Pierce BCA Protein Assay Kit (ThermoFisher Scientific). An aliquot containing a total protein of 200 μg from each individual CMP isolate pool was then transferred to 2-mL microcentrifuge tubes (VWR, Radnor, PA) and stored at − 80 °C pending completion of all CMP isolate processing. All CMP isolates were then shipped on dry ice to BGI Americas Corporation (Cambridge, MA, USA) for proteomic analysis.

### Sample lysis

A total 158 Plasma enriched exosome samples were individually processed for LC–MS/MS analysis. 100 µL of enriched exosomes were mixed with 700 µL of lysis buffer that contained 9 M urea, at pH 8.5, and 0.5% Rapigest (SKU: 186001861, Waters™). Samples were water-bath sonicated for 30 min followed by spinning at high-speed (14,000 rpm) in a centrifuge for 10 min. The protein concentration of samples was measured by the BCA assay (Cat No: A53225, ThermoFisher Scientific) post sample lysis.

### Proteomics sample preparation

50 µg of each sample was taken from the lysate and normalized to the same volume with lysis buffer. Samples were reduced in 10 mM DTT for 25 min at 60 °C, then the reduced samples were alkylated in 20 mM IAM (iodoacetamide) in a dark environment for 20 min at room temperature. Excess IAM in the samples was quenched by adding 100 mM DTT. DI water and HEPE buffer at pH 8.5 were added to each sample so that the final urea concentration was diluted to 1.6 M, and a final pH of 8, for enzymatic digestion. 1 µg of Try/LysC (Cat No: A41007, ThermoFisher Scientific) was added to each sample. The samples were incubated overnight at 37 °C for 12 h. An additional 1 µg of Tryp/LysC was added to each sample the next day and they were incubated for another 4 h to complete the enzymatic digestion.

### Peptide cleanup and fractionation

10% TFA was added into the digested samples (peptides) to produce a final concentration of 1% TFA—the pH was tested and the samples were acidic. Then, acidified samples were passed through a 10 mg SEK PAK column (Cat No: 60108-302, ThermoFisher Scientific) for desalting. 20% of the desalted peptides of each sample was taken and pooled together to create a composite “library” of peptides. The library samples were then fractionated into 96 fractions with a high pH, reverse-phase, offline HPLC fractionator (VanquishTM, ThermoFisher Scientific). The mobile phase A was made up of DI H_2_O with 20 mM formic acetate, pH 9.3; the mobile phase B was made up of acetonitrile (Optima™, LC/MS grade, Fisher Chemical™) with 20 mM formic acetate, pH 9.3. The gradient of separation is displayed in Table S7. 96 fractions were then combined into 24 fractions and readied for liquid chromatography mass spectrometry (LC/MS) analysis.

### LC–MS/MS analysis

All fractionated samples were analyzed by nanoflow HPLC (Ultimate 3000, Thermo Fisher Scientific) followed by Thermo Orbitrap mass spectrometer (Tribrid Eclipse) analysis. A Nanospray Flex™ Ion Source (Thermo Fisher Scientific) was equipped with Column Oven (PRSO-V2, Sonation) to heat up the nanocolumn (PicoFrit, 100 µm × 250 mm × 15 µm tip, New Objective) for peptide separation. The nanoLC method is water acetonitrile based that was 150 min long with a 0.300 µL/min flowrate. For each sample injection, all peptides were first engaged on a trap column (Cat. No: 160454, Thermo Fisher) and then were delivered to the separation nanocolumn by the mobile phase. The specifics of the gradient used are provided in Table S8. For the DDA library construction, a DIA library-specific DDA, MS2-based mass spectrometry method on Eclipse was used to sequence fractionated peptides that were eluted from the nanocolumn. For the full MS spectrum, a resolution of 120,000 was used with a scan range of 375–1500 m/z. For the dd-MS(MS2), a resolution of 15,000 was used, and the isolation window is 1.6 Da. ‘Standard’ AGC target and ‘Auto’ Max Ion injection times (Max IT) were selected for both MS1 and MS2 acquisition. The collision energy (NCE) was set to 35%, and the total cycle time is 1 s. For DIA analytical samples, a high-resolution, full MS scan, followed by two segment DIA methods, was used for the DIA data acquisition. For the full MS scan, a resolution of 120,000 was used for the range of 400–1200 m/z with a ‘Standard’ AGC target and 50 ms Max IT. For both DIA segments, the details of the isolation windows (IW) and the precursor mass ranges are shown in Table S9 and S10. For the DIA fragments scan, a resolution of 30,000 was used for the range of 110–1800 m/z with a ‘Standard’ AGC target and ‘Auto’ Max IT.

### Bioinformatic analysis pipeline overview

The initial process is based on the sample data generated from a high-resolution mass spectrometer. The DDA data was identified by the Andromeda search engine within MaxQuant, and Spectronaut™ was used for the identification of results for spectral library construction. MaxQuant was used for the identification of DDA data, which served as a spectrum library for the subsequent DIA analysis. The analysis pipeline used raw data as input files and set corresponding parameters and human databases (UP000005640), then the identification and quantitative analysis was performed. The identified peptides satisfied a FDR of ≤ 1% to construct the final spectral library. For this DIA dataset, Spectronaut™ was employed to construct spectral library information to complete deconvolution and extraction, then the mProphet algorithm was used to complete an analytical quality control (1% FDR) to obtain reliable quantitative results. GO, COG, and Pathway functional annotation analysis and time series analysis were also performed in the pipeline described above. MStats, the core algorithm of which is a linear mixed effect model, was used to process the DIA quantification results data according to the predefined comparison group, and then a significance test was performed based on the model. Thereafter, differential protein screening was executed and a fold change of ≥ 2 and an adj P-value of < 0.05 was defined as a significant difference. Based on the quantitative comparison results, the differential proteins between comparison groups were identified; finally a function enrichment analysis, a protein–protein interaction (PPI) examination, and a subcellular localization analysis of the differential proteins were carried out. The sample classification analyses were then implemented as described below.

### Sample classification

We implemented a two-step workflow described in a prior publication^50^*.* PAS was classified using regularized (L1) regression to define a restricted set of candidate CMP proteins from the superset of all identified proteins. To select our putative panel from the restricted set of candidate CMP proteins, we chose a cross-validation procedure using logistic regression. The logic of this workflow was adapted from that of Yoffe et al.^53^. The sample was randomly divided into a training and validation set (80% vs. 20%). The proteins in the training set were then ranked by their Akaike information criterion (AIC) using an ensemble feature selection procedure^54^. The top 10 proteins were then passed to the *glmulti* package in R version 3.6.3 where the training set was subjected to fivefold cross-validation^55^. To avoid overfitting, given the limited sample size, the model was restricted to no more than 5 predictors. The model with the greatest area under the curve (AUC) and the lowest standard deviation of the AUC was then tested against the set-aside, external validation set. The AUC and standard deviation of the AUC of this external validation set was then recorded and the workflow re-iterated for a total of 1000 iterations (Fig. S1). The models were then ranked by their mean AUC and mean standard deviation of the AUC. The workflow was then repeated with randomly permuted sample labels. Predictive statistics for the observed versus permuted data were then compared^16,17^*.*

### Ingenuity pathway analysis

To determine the biological function of those proteins identified as differentially expressed by PAS status in our differential expression analysis, we also applied Ingenuity Pathway Analysis (IPA, version 01-20-04, run April of 2022). Core analysis with the Ingenuity Knowledge Base reference set was used. Direct and indirect relationships were considered for network and regulatory analyses. IPA is a curated bioinformatic repository of functionally annotated analytes which allows for functional annotation, canonical pathway and network analyses, and upstream regulator analysis. Significantly (P < 0.05) over-represented canonical pathways, upstream regulators, and molecular and cellular functions were identified. Only relevant pathways and biological functions containing two or more overlapping hits were included, while only upstream regulators with a predicted activation state (activated, inhibited) were included. Overlap ratios were calculated as the percent of overlap between differentially expressed proteins and the target pathway.

## Supplementary Information

Supplementary Information.

## Data Availability

Data are available with a signed data use agreement to protect identifiable data; please contact hyu20@bwh.harvard.edu.

## Code availability

Code is available upon request; please contact hyu20@bwh.harvard.edu.
