# Supplementary material for: Circulating microparticle proteins predict pregnancies complicated by placenta accreta spectrum
Source: Sci Rep. 2023 Jan 5;12:21922. doi: 10.1038/s41598-022-24869-0 (PMC9814521; doi:10.1038/s41598-022-24869-0)
Supplement: Supplementary file 1 — Supplementary Information. [file 41598_2022_24869_MOESM1_ESM.pdf]

Supplementary Materials for

Circulating microparticle proteins predict pregnancies complicated by placenta accreta spectrum

Hope Y Yu MD[1]*; Serena B Gumusoglu PhD[2]; David E Cantonwine PhD[1]; Daniela A Carusi MD MSc[1]; Prem Gurnani MS[3]; Brandon Schickling PhD[2]; Robert C Doss PhD[3]; Mark K Santillan MD PhD[2]; Kevin P Rosenblatt MD PhD[3,4**]; Thomas F McElrath MD PhD[1**]

[1]Division of Maternal-Fetal Medicine, Department of Obstetrics and Gynecology, Brigham and Women's Hospital, Boston, MA 02115, USA.
[2]University of Iowa Carver College of Medicine, Iowa City, IO, USA.
[3]NX Prenatal Inc., Louisville KY, USA.
[4]Division of Oncology, Department of Internal Medicine, University of Texas Health Science center at Houston, McGovern Medical School, Houston, TX, USA.

*Corresponding author. Email: hyu20@bwh.harvard.edu

**Authors contributed equally

This PDF file includes:

Tables S1 to S10

Ingenuity Canonical Pathways	-log(p-value)	Overlap ratio	Molecules
Erythropoietin Signaling Pathway	4.33	0.0169	HBA1/HBA2,HBB,HBG1
Iron homeostasis signaling pathway	4.64	0.0216	HBA1/HBA2,HBB,HBG1

Table S1. Canonical pathways significantly over-represented by differentially expressed proteins in second trimester placenta accreta cases.

Master Regulator	Molecule Type	Predicted Activation State	p-value of overlap	Target molecules in dataset
signal peptidase	complex	Activated	0.0000173	ALB,ANGPTL2,GRN,HBA1/HBA2,HBB,NID1,VNN1
GNRHR	G-protein coupled receptor	Activated	0.000143	GRN,HBA1/HBA2,HBB,VNN1
DRD3	G-protein coupled receptor	Activated	0.000148	GRN,HBA1/HBA2,HBB,VNN1
ARAF	kinase	Activated	0.000408	GRN,HBA1/HBA2,HBB,VNN1
G protein alpha	group	Activated	0.000471	GRN,HBA1/HBA2,HBB,VNN1
F10	peptidase	Activated	0.000649	ANGPTL2,HBA1/HBA2,HBB,VNN1
BDKRB2	G-protein coupled receptor	Activated	0.000826	GRN,HBA1/HBA2,HBB,VNN1
MAP2K7	kinase	Inhibited	0.000139	ALB,ANGPTL2,HBA1/HBA2,HBB,HBG1
CDK5R1	kinase	Inhibited	0.000269	ANGPTL2,GRN,HBA1/HBA2,HBB,VNN1
SERPINB1	other	Inhibited	0.000691	ANGPTL2,GRN,HBA1/HBA2,HBB,NID1,VNN1

Table S2. Select master upstream regulators of target molecules in second trimester placenta accreta dataset.

Diseases or Functions Annotation	p-value	Molecules
Heinz body anemia	1.59E-07	HBA1/HBA2,HBB
Mineral deficiency	2.90E-07	ALB,HBA1/HBA2,HBB
Iron deficiency anemia of pregnancy	4.77E-07	HBA1/HBA2,HBB
Refractory iron deficiency	4.77E-07	HBA1/HBA2,HBB
Hereditary persistence of fetal hemoglobin	1.59E-06	HBB,HBG1
Transport of oxygen	2.38E-06	HBA1/HBA2,HBB
Biosynthesis of hydrogen peroxide	1.99E-05	ALB,HBA1/HBA2,HBB
Catabolism of hydrogen peroxide	3.01E-05	HBA1/HBA2,HBB
Hemolysis	3.59E-05	ALB,HBA1/HBA2,HBB
Degranulation of phagocytes	9.34E-05	ALB,GRN,HBB,VNN1
Degranulation of myeloid cells	9.86E-05	ALB,GRN,HBB,VNN1
Familial hemolytic anemia	1.29E-04	HBA1/HBA2,HBB
Peripheral vascular disease	1.49E-04	ALB,GRN,HBA1/HBA2,HBB
Aggregation of blood platelets	1.60E-04	ALB,ANGPTL2,HBB
Polycythemia	1.90E-04	GRN,HBA1/HBA2,HBB
Receptor-mediated endocytosis	2.10E-04	ALB,HBA1/HBA2,HBB
Synthesis of reactive oxygen species	2.30E-04	ALB,GRN,HBA1/HBA2,HBB
Quantity of reticulocytes	3.26E-04	HBA1/HBA2,HBB
Endocytosis	4.02E-04	ALB,GRN,HBA1/HBA2,HBB
Degranulation of neutrophils	9.46E-04	GRN,HBB,VNN1
Formation of epithelial tissue	9.64E-04	GRN,NID1
Hypertension	1.02E-03	ALB,ANGPTL2,HBA1/HBA2,HBG1
Ischemic stroke	1.11E-03	ALB,GRN
Production of reactive oxygen species	1.54E-03	GRN,HBA1/HBA2,HBB
Production of superoxide	1.95E-03	HBA1/HBA2,HBB
Quantity of myeloid cells	5.34E-03	GRN,HBA1/HBA2,HBB
Consumption of oxygen	5.88E-03	HBA1/HBA2,HBB
Cell movement of myeloid cells	5.88E-03	ALB,ANGPTL2,GRN
Generation of reactive oxygen species	6.20E-03	ALB,HBA1/HBA2
Innate immune response	7.24E-03	GRN,VNN1
Coagulation of blood	1.01E-02	HBB,HBG1
Severe inflammatory disorder	1.05E-02	GRN,HBG1
Synthesis of nitric oxide	1.18E-02	ALB,ANGPTL2
Inflammatory response	1.48E-02	ANGPTL2,GRN,VNN1
Cell movement of neutrophils	1.73E-02	ALB,GRN
Apoptosis	2.49E-02	ALB,GRN,HBA1/HBA2,HBB,VNN1
Quantity of metal ion	2.73E-02	GRN,HBA1/HBA2
Abnormal morphology of epithelial tissue	2.75E-02	GRN,NID1
Bleeding	2.95E-02	ALB,GRN
Development of vasculature	3.62E-02	ANGPTL2,GRN,NID1
Preeclampsia	3.73E-02	ANGPTL2,HBG1
Chronic inflammatory disorder	3.77E-02	ALB,GRN,HBA1/HBA2
Development of epithelial tissue	4.45E-02	GRN,NID1

Table S3. Select over-represented molecular and cellular functions of proteins altered in second trimester placenta accreta

Ingenuity Canonical Pathways	-log(p-value)	Overlap Ratio	Molecules
Communication between Innate and Adaptive Immune Cells	2.9	0.00431	IGHV3-20,IGKV3D-11,IGKV4-1,IGLV2-18
B Cell Receptor Signaling	3.54	0.00634	IGHV3-20,IGKV3D-11,IGKV4-1,IGLV2-18
Integrin Signaling	3.72	0.0141	ARPC5,ILK,PARVB
IL-15 Signaling	3.84	0.00758	IGHV3-20,IGKV3D-11,IGKV4-1,IGLV2-18

Table S4. Select canonical pathways significantly over-represented by differentially expressed proteins in third trimester placenta accreta cases.

Master Regulator	Molecule Type	Predicted Activation State	p-value of overlap	Target molecules in dataset
DVL1	other	Activated	0.000188	GAPDH,HSPG2,ILK,MPO,PARVB
Histone h3	group	Activated	0.00121	ARPC5,GAPDH,HSPG2,ILK,MPO
ADCY	group	Activated	0.00168	ARPC5,GAPDH,HSPG2,PARVB
RGS (2,4,7,16,18)	group	Inhibited	0.0000907	GAPDH,HSPG2,ILK,MPO,SPP2

Table S5. Select master upstream regulators of target molecules in third trimester placenta accreta dataset.

Diseases or Functions Annotation	p-Value	Molecules
Classical complement pathway	0.0000102	IGKV4-1,C1QB,IGKV3D-11
Receptor-mediated endocytosis	0.000487	IGKV4-1,HSPG2,IGKV3D-11
Cell movement	0.00278	IGKV4-1,MPO,HSPG2,GAPDH,IGKV3D-11,PARVB,ILK
Reorganization of actin cytoskeleton	0.00342	PARVB,ILK
Attachment of cells	0.0035	HSPG2,ILK
Apoptosis of cardiomyocytes	0.00764	MPO,GAPDH
Cellular infiltration	0.0079	MPO,GAPDH,ILK
Leukocyte migration	0.0087	IGKV4-1,MPO,IGKV3D-11,ILK
Migration of connective tissue cells	0.00895	HSPG2,ILK
Aggregation of blood platelets	0.00908	MPO,ILK
Degranulation of cells	0.00916	SPP2,ARPC5,MPO
DNA damage	0.0159	MPO,GAPDH
Organization of cellular membrane	0.0165	ARPC5,ILK
Cell death of epithelial cell lines	0.0213	MPO,GAPDH
Cell death of kidney cell lines	0.0213	GAPDH,ILK
Familial heart disease	0.0242	HSPG2,ILK
Acute respiratory disorder	0.0258	MPO,GAPDH
Immune mediated inflammatory disease	0.0267	MPO,C1QB,GAPDH,ILK
Degranulation of neutrophils	0.0286	ARPC5,MPO
Cell movement of neutrophils	0.0288	MPO,ILK
Adhesion of immune cells	0.0305	MPO,ILK
Organization of cytoskeleton	0.0322	ARPC5,GAPDH,PARVB,ILK
Disorder of pregnancy	0.0337	MPO,GAPDH,ILK
Migration of cells	0.0376	IGKV4-1,MPO,HSPG2,IGKV3D-11,ILK

Table S6. Select over-represented molecular and cellular functions of proteins altered in third trimester placenta accreta

Time[min]	Flow[ml/min]	%B
0.00	0.500	2.0
1.00	0.500	6.0
12.00	0.500	20.0
30.00	0.500	28.0
50.00	0.500	65.0
53.00	0.500	98.0
57.00	0.500	98.0
59.00	0.500	2.0
60.00	0.500	2.0

Table S7. High-pH, Reverse-Phase HPLC Fractionation Gradient Information

Time[min]	Flow[µl/min]	%B
0.00	0.300	2.0
3.00	0.300	2.0
3.10	0.300	2.0
8.00	0.300	4.0
98.00	0.300	35.0
128.00	0.300	65.0
129.00	0.300	100.0
133.00	0.300	100.0
134.00	0.300	2.0
140.00	0.300	2.0
150.00	0.300	2.0

Table S8. nanoLC-MS Gradient Information.

**Segment 1 (400 – 800 m/z, IW 15 m/z, Overlap 1 m/z)**	
399.5 – 415.5	609.5 – 625.5
414.5 – 430.5	624.5 – 640.5
429.5 – 445.5	639.5 – 655.5
444.5 – 460.5	654.5 – 670.5
459.5 – 475.5	669.5 – 685.5
474.5 – 490.5	684.5 – 700.5
489.5 – 505.5	699.5 – 715.5
504.5 – 520.5	714.5 – 730.5
519.5 – 535.5	729.5 – 745.5
534.5 – 550.5	744.5 – 760.5
549.5 – 565.5	759.5 – 775.5
564.5 – 580.5	774.5 – 790.5
579.5 – 595.5	789.5 – 800.5
594.5 – 610.5	

Table S9. DIA segment 1 Precursor Scan Range Information.

**Segment 2 (800 – 1200 m/z, IW 25 m/z, Overlap 1 m/z)**	
799.5 – 825.5	999.5 – 1025.5
824.5 – 850.5	1024.5 – 1050.5
849.5 – 875.5	1049.5 – 1075.5
874.5 – 900.5	1074.5 – 1100.5
899.5 – 925.5	1099.5 – 1125.5
924.5 – 950.5	1024.5 – 1150.5
949.5 – 975.5	1049.5 – 1175.5
974.5 – 1000.5	1074.5 – 1200.5

Table S10. DIA segment 2 Precursor Scan Range Information.

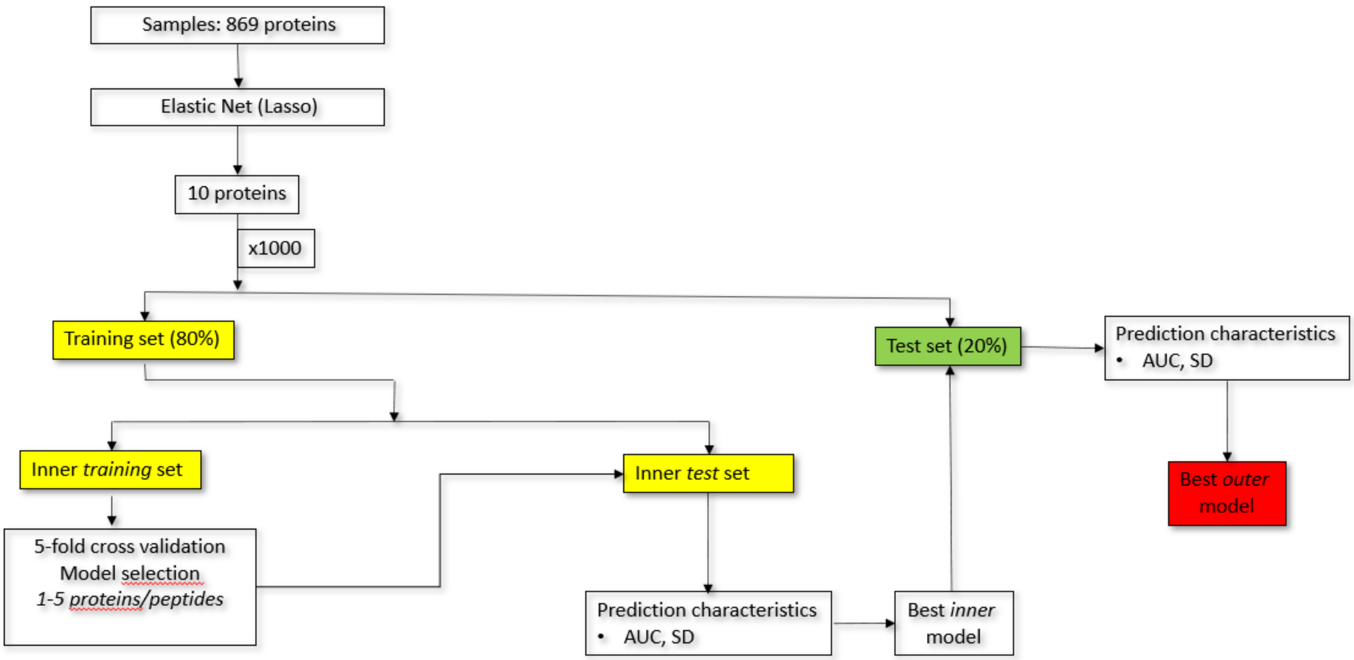

Figure S1. Schematic of workflow for case versus control CMP protein identification.
